# Conditional diffusion-based inverse kinematics for redundant 7-DOF manipulators with differentiable refinement

**DOI:** 10.3389/frai.2026.1884749

**Published:** 2026-10-16

**Authors:** Lesia Hentosh, Yaroslav Hladun, Ivan Tsmots

**Affiliations:** Department of Artificial Intelligence Systems, Lviv Polytechnic National University, Lviv, Ukraine

**Keywords:** classifier-free guidance, conditional diffusion models, DDIM sampling, differentiable forward kinematics, generative robotics, inverse kinematics, redundant manipulators

## Abstract

**Background:**

Inverse kinematics of redundant manipulators is a multi-solution problem, since one end-effector pose may correspond to multiple admissible joint configurations. Classical numerical solvers and regression-based neural models usually return a single joint configuration and therefore do not explicitly represent the inverse-kinematic solution manifold. Existing generative solvers based on normalizing flows produce multiple solutions, but they require invertible network architectures and do not include an explicit task-space correction stage. This study aimed to develop a hybrid generative-kinematic method for inverse kinematics of a redundant 7-DOF manipulator that can generate multiple admissible configurations for a given target pose.

**Methods:**

A hybrid method, DiffusionIK, was developed by combining conditional diffusion-based generation, classifier-free guidance, DDIM sampling, and differentiable forward-kinematics refinement. A ResMLP denoiser is trained in the normalized joint space using procedurally generated training pairs obtained from the forward kinematics of the xArm manipulator. For each target pose, the diffusion model generated multiple candidate joint configurations, and the refinement stage subsequently reduced the task-space residual for each candidate. Numerical experiments were performed on 500 reachable target poses with 50 generated candidates per pose. Performance was evaluated using position and orientation success thresholds of 1 mm and 1°, respectively, together with the preservation of joint-space variability after refinement.

**Results:**

DiffusionIK reached 99.40% success after 200 refinement steps and 100.00% after 500 steps under 1 mm and 1° thresholds, with a mean joint-space variability of 9.77 rad. In a unified benchmark against damped least squares, KDL, direct regression, and a normalizing-flow baseline, DiffusionIK with refinement achieved 99.80% success on 500 unseen poses and 98.40% near singularities, matching the strongest numerical solver while returning 50 diverse solutions per pose.

**Conclusion:**

The proposed DiffusionIK method provides an effective formulation of redundant inverse kinematics as conditional generation followed by local kinematic correction. By combining diffusion-based candidate generation with differentiable forward-kinematics refinement, the method represents the multi-solution nature of redundant manipulators and achieves high task-space accuracy while preserving configuration diversity. Unlike normalizing-flow-based solvers, DiffusionIK imposes no invertibility constraints on the denoiser and combines generation with differentiable forward-kinematics refinement, which provides sub-millimetre and sub-degree accuracy together with configuration diversity.

## 1 Introduction

Inverse kinematics (IK) is one of the fundamental problems in robotics (Sanz, 2009). It consists in determining the joint configuration of a manipulator for a given end-effector pose. Let $Q\subset {\mathbb{R}}^{n}$ denote the admissible configuration space, and let forward kinematics (FK), FK:Q→SE(3) , define the mapping from the joint space to the task space. Here, the Special Euclidean group in three dimensions (SE(3)) represents rigid-body positions and orientations. For a target pose $y^{*}=(R^{*},t^{*})$ , where $R^{*}\in \mathrm{SO}(3)$ , the Special Orthogonal group in three dimensions (SO(3)) represents rotations, and $t^{*}\in {\mathbb{R}}^{3}$ , the IK problem is to find a joint vector q∈Q such that

$$\mathrm{FK}(q)=y^{*}.$$

For redundant manipulators, this problem does not have a unique solution. If n>6 , the same end-effector pose may correspond to multiple joint configurations. The local dimensionality of this solution set can be expressed as

$$\dim\;S(y^{*})=n-\mathrm{rank}J_{\mathrm{FK}}(q),$$

where $J_{\mathrm{FK}}(q)$ is the FK Jacobian, and

$$S(y^{*})=\{q\in Q:\mathrm{FK}(q)=y^{*}\}$$

denotes the set of kinematically admissible solutions for the target pose. For a seven-degree-of-freedom (7-DOF) manipulator with a full-rank Jacobian, this set forms a one-dimensional self-motion manifold. This property distinguishes IK for redundant manipulators from a conventional regression problem.

Classical numerical IK methods usually construct a single solution. These methods include Jacobian-based approaches, damped least squares, Cyclic Coordinate Descent (CCD), and Forward And Backward Reaching Inverse Kinematics (FABRIK). The damped least-squares method improves numerical stability near singular configurations; however, the obtained solution remains dependent on the initial approximation and the damping parameter (Nakamura and Hanafusa, 1986). FABRIK performs iterative adjustment of link positions in Cartesian space, but it does not explicitly represent the solution manifold of redundant manipulators (Aristidou and Lasenby, 2011). Therefore, classical methods are local solvers. They provide one kinematically admissible configuration but do not recover the set of alternative configurations that may be required for subsequent motion planning.

Machine learning methods have enabled the approximation of complex nonlinear dependencies between the end-effector pose and joint angles. However, the direct regression formulation $f_{\theta }:\mathrm{SE}(3)\to Q$ is limited for redundant manipulators. It transforms a problem with multiple admissible outputs into a single-output prediction task. In the presence of a multimodal solution distribution, such a formulation may lead to averaging across different modes. As a result, the model may generate a configuration that does not reflect the actual structure of $S(y^{*})$ . Therefore, redundant kinematic systems require a distributional formulation rather than a pointwise approximation.

Generative models provide a natural framework for such a formulation because they make it possible to model the conditional distribution $p(q|,R^{*}|,t^{*})$ and sample a set of configurations from it. One of the closest approaches to this idea is IKFlow, where normalizing flows are used to generate diverse IK solutions (Ames et al., 2022). This approach demonstrated the relevance of moving from a single solution to a set of kinematic candidates. At the same time, normalizing flows require invertible transformations. This restricts the model architecture and complicates the use of arbitrary residual blocks, normalization layers, and nonlinear transformations. Therefore, the construction of a generative IK solver that preserves the multimodality of the configuration space and does not require an invertible architecture remains an open problem.

Diffusion models provide a formal basis for addressing this problem. They learn to recover data from progressively noised states and can approximate complex multimodal distributions (Ho et al., 2020). Denoising Diffusion Probabilistic Models (DDPM) define an iterative denoising process for data reconstruction from noise. Denoising Diffusion Implicit Models (DDIM) reduce the number of generation steps compared with the full Markovian DDPM scheme (Song et al., 2020). Classifier-Free Guidance (CFG) strengthens the influence of the conditioning input on the generated output without training a separate classifier (Ho and Salimans, 2022). In robotics, diffusion models have already been applied to policy learning, motion planning, and trajectory generation (Carvalho et al., 2023; Chi et al., 2025). Recent journal studies have also begun to examine diffusion-augmented formulations for IK. However, the coordinated integration of conditional diffusion generation, guidance-based pose conditioning, and differentiable FK refinement for redundant 7-DOF manipulators remains insufficiently explored (Wang et al., 2026). The computational aspect is also important for robotic systems operating under real-time constraints. In previous work, CUDA-based parallelization of the Kalman localization algorithm for LiDAR data was shown to reduce the computational time of 3D localization while preserving localization accuracy (Mochurad, 2024).

This work proposes DiffusionIK for solving IK of a 7-DOF manipulator. The method formulates the problem not as the search for a single vector $q^{*}$ , but as the generation of a set of candidates

$${\left\{q_{i}\right\}}_{i=1}^{M}∼p_{\theta }(q\;|\;R^{*},t^{*}).$$

Each candidate is refined according to a kinematic criterion through differentiable FK. For this purpose, the residual in the task space is minimized as

$$L_{\mathrm{FK}}(q)={\left\|t(\mathrm{FK}(q))-t^{*}\right\|}_{2}^{2}+\lambda {\left\|R(\mathrm{FK}(q))-R^{*}\right\|}_{F}^{2}.$$

This scheme separates two subproblems. The diffusion model performs global generation of diverse initial configurations. Gradient refinement provides local reduction of the kinematic error for each candidate.

The aim of this work is to develop a generative-kinematic IK method for a redundant 7-DOF manipulator that forms a set of diverse admissible joint configurations for one target end-effector pose and further refines them in SE(3) . In contrast to deterministic numerical solvers and regression-based neural models, the proposed formulation is focused on approximating the conditional distribution of solutions rather than constructing a single prediction.

The main contribution of this work is the development of DiffusionIK, a hybrid generative-kinematic method for the IK problem of a redundant 7-DOF manipulator. The method combines conditional diffusion-based sampling, CFG, DDIM sampling, and gradient refinement through differentiable FK in a unified computational scheme. In the proposed scheme, the diffusion model forms a set of initial configurations in the joint space, while kinematic refinement brings each configuration into agreement with the target end-effector pose. The main results of the work are as follows:

- A hybrid generative-kinematic IK method has been developed for a redundant 7-DOF manipulator. In the proposed method, a conditional diffusion model generates a set of diverse initial joint configurations, while differentiable FK refines these configurations in the task space. In contrast to direct regression models, the method does not reduce IK to a single deterministic prediction but represents it as the generation and refinement of multiple admissible configurations on the solution manifold.
- The generative formulation of the IK problem has been improved by integrating diffusion-based sampling, DDIM sampling, and CFG. This integration strengthens the consistency of the generated configurations with the target end-effector pose while preserving variability in the joint space. Unlike normalizing-flow-based IK methods, the proposed formulation does not require an invertible architecture.
- The architectural implementation of the generative IK solver has been improved by using a Residual Multi-Layer Perceptron denoiser (ResMLP) combined with differentiable FK refinement. The ResMLP denoiser models dependencies between the joint configuration, the target pose, and the diffusion step, whereas the refinement stage reduces the kinematic residual for each generated candidate. This provides a coordinated combination of a learned generative prior and kinematic verification for obtaining multiple admissible configurations for one target pose.

The practical value of the proposed method lies in the ability to obtain not one but a set of diverse kinematically admissible solutions for a single target pose. Such configurations can be used for the subsequent selection of a manipulator posture with regard to joint limits, singular configurations, self-collisions, obstacles in the workspace, and requirements imposed by the next motion. This creates a basis for using the method as a generator of initial configurations in motion planning, as a module for searching alternative manipulator postures, and as a foundation for further extension to trajectory generation tasks. At the same time, the availability of several admissible configurations does not by itself guarantee the feasibility of an arbitrary secondary task. For the studied 7-DOF manipulator executing a full 6-DOF task, a single redundant degree of freedom is available, and in dynamic environments the number of additional degrees of freedom required by the complete task cannot be established *a priori*. The generated set of configurations should therefore be regarded as an enabling component for downstream posture selection and motion planning rather than as a guarantee that all additional constraints can be satisfied.

## 2 State-of-the-art

The inverse kinematics problem has a long history of research in robotics. Its classical formulation is associated with finding a joint configuration that enables the end-effector to reach a specified pose. For non-redundant manipulators, this problem may have a finite number of solutions or admit an analytical solution for specific kinematic structures. For redundant manipulators, the situation is more complex, since one target pose may correspond to a set of admissible configurations in the joint space. This property necessitates not only the local computation of a single solution but also the modeling of a set of possible configurations.

Classical numerical methods for inverse kinematics are based on local linearization of the kinematic mapping. This line of methods originates from resolved-rate control and Jacobian-based manipulator control formulations (Campa et al., 2010). The pseudoinverse Jacobian method and the damped least-squares method directly use the relationship between the increment of the joint configuration and the error in the task space. Damping improves stability near singular configurations; however, it does not eliminate the dependence of the result on the initial approximation and regularization parameters (Nakamura and Hanafusa, 1986). Therefore, these methods are effective local solvers, but they do not form a distribution of admissible configurations for a single target pose. The use of regularization is not specific only to inverse kinematics; it is a general mechanism for stabilizing ill-conditioned inverse computational problems. For example, Tikhonov regularization combined with OpenMP-based parallel execution has been applied to Fredholm integral equations of the first kind (Mochurad et al., 2020).

Heuristic methods, in particular Forward And Backward Reaching Inverse Kinematics and Cyclic Coordinate Descent, reduce computational complexity and do not require explicit computation of the Jacobian. Related combined optimization formulations were also proposed for solving inverse kinematics of mechanical manipulators (Wang and Chen, 1991). Forward And Backward Reaching Inverse Kinematics sequentially adjusts the positions of the links in Cartesian space, which makes it suitable for rapid refinement of the end-effector position (Aristidou and Lasenby, 2011). At the same time, such methods do not explicitly represent the solution manifold of a redundant manipulator. They return a single configuration rather than a set of alternative postures that could be further selected with regard to constraints, obstacles, or the subsequent motion.

Modern optimization approaches to redundant inverse kinematics extend classical numerical methods by explicitly accounting for constraints. In particular, Zhang et al. (2024) proposed a real-time method for redundant robots with inequality constraints in both the joint space and Cartesian space. The authors emphasize that inverse kinematics for redundant robots is complicated by the non-uniqueness of the solution and by strict bounds that must not be violated during motion. This direction is important for practical control. However, it remains optimization-based and is oriented toward selecting a single admissible solution under given conditions. It does not consider the generation of a set of candidates as a separate task.

Machine learning methods have enabled a transition from explicit iterative optimization to data-driven approximation of kinematic dependencies. Recent reviews show the active development of deep neural networks for inverse kinematics, control, and motion planning of robotic manipulators (Calzada-Garcia et al., 2025a). Neural network models can reduce inference time after training and can capture nonlinear kinematic relationships. However, most of these approaches retain a regression-based formulation. The model is trained to predict a single joint vector for a given end-effector pose. For redundant manipulators, this is a fundamental limitation, because a multi-solution problem is reduced to a single prediction. Earlier neural-network and soft-computing approaches demonstrated the feasibility of data-driven inverse kinematics approximation, but they mainly retained a single-output formulation (Duka, 2014; El-Sherbiny et al., 2018).

Experimental studies of deep learning approaches to inverse kinematics confirm that different types of architectures can approximate complex kinematic mappings. Earlier experimental studies also examined neural-network-based inverse kinematics for articulated robots (Csiszar et al., 2017). Studies published in 2023–2025 have considered multilayer neural networks, convolutional models, recurrent architectures, liquid neural networks, and graph-based models for robotic manipulators (Calzada-Garcia et al., 2025b; Cagigas-Muñiz, 2023; Wagaa et al., 2023; Manjegowda and Rao, 2025; Zhang et al., 2025). These studies expand the methodological toolkit for neural-network-based inverse kinematics. At the same time, their primary objective is usually to improve the accuracy of a single prediction or to reduce computation time. The problem of generating or representing a set of admissible configurations for a single end-effector pose remains insufficiently addressed.

The regression-based formulation has another limitation. In the case of a multimodal distribution of solutions, minimizing the mean squared error may lead to averaging across different modes. Such an averaged joint vector does not necessarily belong to the set of kinematically admissible configurations. This is especially important for redundant manipulators, where different configurations may correspond to the same end-effector pose. Therefore, this problem requires not only the approximation of the mapping from pose to joints but also the modeling of the conditional distribution of configurations. Generative adversarial models have also been applied to learning inverse kinematics and dynamics of robotic manipulators (Ren and Ben-Tzvi, 2020). However, such models may suffer from unstable training and mode collapse, which makes them less direct for representing the complete solution manifold of redundant inverse kinematics.

Generative approaches are more consistent with the nature of redundant inverse kinematics. One of the closest approaches to this idea is IKFlow, in which normalizing flows are used to generate diverse inverse kinematics solutions (Ames et al., 2022). This approach has shown that, for systems with more than six degrees of freedom, it is appropriate to generate a set of configurations, since a single target pose may have infinitely many solutions. IKFlow represents an important step from pointwise prediction to a distributional formulation. At the same time, normalizing flows require invertible transformations. This imposes architectural constraints and complicates the use of arbitrary residual blocks, normalization layers, and nonlinear transformations.

Diffusion models offer a different generative paradigm. They do not require the construction of an explicit invertible mapping between the base distribution and the target distribution. Instead, the model learns to progressively recover data from a noisy state. This property is important for problems with a multimodal space of admissible outputs. In robotics, diffusion models are already used for policy learning, motion planning, and trajectory generation. Diffusion-based trajectory generation has also been studied in the context of flexible behavior synthesis and planning (Janner et al., 2022). However, these tasks are not identical to inverse kinematics. In these cases, the object of modeling is an action, a trajectory, or a cost function, rather than a set of joint configurations for a single given pose.

Diffusion Policy formulates a robotic policy as a conditional diffusion process over actions. The journal version of this work shows that a diffusion-based representation makes it possible to handle multimodal action distributions in manipulation tasks (Chi et al., 2025). This supports the suitability of diffusion models for robotic problems with ambiguous outputs. However, Diffusion Policy does not solve the geometric problem of inverse kinematics. It does not generate a set of joint configurations for a given end-effector pose.

Motion Planning Diffusion uses a diffusion model as a prior for motion planning. In this work, the diffusion distribution is used to encode trajectories and support optimization-based planning, particularly in environments with obstacles (Carvalho et al., 2023). This shows that diffusion models can be useful for high-dimensional distributions in robotics. However, the object of modeling remains a trajectory rather than the conditional distribution of configurations for a fixed end-effector pose.

Another research direction concerns diffusion models in the space of spatial transformations. SE(3)-DiffusionFields uses a diffusion-based approach to learn smooth cost functions for joint grasping and motion planning tasks (Urain et al., 2023). This direction supports the relevance of using diffusion processes in spaces related to position and orientation. At the same time, it does not formulate the problem as the generation of a set of joint configurations for a given end-effector pose.

Recent studies have begun to examine diffusion-augmented approaches to inverse kinematics. In particular, Wang et al. (2026) proposed a hybrid method for real-time inverse kinematics, in which a diffusion component is combined with optimization-based and neural network modules. This confirms the relevance of diffusion-based approaches for IK problems. However, in this formulation, diffusion is used as an auxiliary component of the hybrid scheme. It is not a direct conditional generator of a set of initial configurations with subsequent refinement of each candidate through differentiable forward kinematics.

Differentiable kinematics is an important component of modern optimization methods for robotic systems. It enables the computation of gradients of kinematic residuals by means of automatic differentiation. This eliminates the need for manual derivation of derivatives and makes it possible to refine the configuration directly according to the error in the task space. Libraries such as Pinocchio and Theseus demonstrate that differentiable modeling and nonlinear optimization are suitable for complex robotic tasks (Carpentier et al., 2019; Pineda et al., 2022). However, differentiable kinematics alone does not provide global coverage of the solution manifold. It is a local refinement mechanism and requires a meaningful initialization.

The reviewed studies leave an unresolved methodological issue: how to generate several pose-conditioned joint configurations and refine each of them without collapsing the set to a single solution. Classical numerical methods provide local convergence but do not form a set of alternative configurations. Constrained optimization methods make it possible to account for joint limits and Cartesian constraints, but they are usually aimed at selecting a single solution. Neural-network regression models reduce inference time, but they reduce a multi-solution problem to a single prediction. Normalizing flows generate diverse IK solutions, but they require an invertible architecture. Diffusion models have been successfully applied to policies, trajectories, and cost functions, but their coordinated use for generating and refining a set of configurations of a redundant manipulator remains insufficiently investigated.

The comparative positioning of the reviewed families of methods with respect to the requirements of redundant inverse kinematics is summarized in Table 1.

**Table 1:** Comparative summary of state-of-the-art approaches to inverse kinematics of redundant manipulators.

Approach	Representative works	Solutions per pose	Invertibility required	Task-space correction	Key limitation
Jacobian-based, damped least squares	Nakamura and Hanafusa (1986)	One (local)	No	Iterative, local	Depends on the initial approximation; single solution
Heuristic iterative (FABRIK, CCD)	Aristidou and Lasenby (2011), Wang and Chen (1991)	One	No	Iterative, local	No representation of the solution manifold
Constrained optimization	Zhang et al. (2024)	One	No	Within the solver	Selects a single constraint-consistent solution
Direct neural regression	Duka (2014), El-Sherbiny et al. (2018), Calzada-Garcia et al. (2025b)	One	No	None	Mode averaging on multimodal solution sets
GAN-based generation	Ren and Ben-Tzvi (2020)	Several	No	None	Unstable training; risk of mode collapse
Normalizing flows (IKFlow)	Ames et al. (2022)	Many	Yes	None	Invertibility requirement constrains the architecture
Diffusion-augmented hybrid	Wang et al. (2026)	one	No	Optimization module	Diffusion is an auxiliary component; single output
DiffusionIK (this work)	—	Many (M per pose)	No	Differentiable FK refinement of every candidate	Collision constraints not yet included

In this article, the identified gap is addressed through the development of DiffusionIK. The method combines conditional diffusion-based generation of initial configurations, classifier-free guidance, DDIM sampling, and gradient refinement through differentiable forward kinematics. In contrast to regression models, it does not form a single deterministic joint vector. In contrast to normalizing flows, it does not require an invertible architecture. In contrast to purely optimization-based solvers, it starts refinement from a set of generated candidates distributed in the configuration space. This provides a methodological transition from the local search for a single solution to the generation and refinement of a set of admissible configurations on the solution manifold.

## 3 Problem formulation

In the general case, a serial manipulator with *n* degrees of freedom is described by a configuration vector *q* ∈ *Q* ⊂ ℝ^n^, where *Q* is the admissible configuration space, and by the forward-kinematics mapping FK: *Q* → SE(3). For a full six-dimensional task, the inverse problem consists in finding *q* such that FK(*q*) = y^*^ for a given target pose y^*^ ∈ SE(3). If *n* > 6, the manipulator is kinematically redundant, and in the regular case of a full-rank Jacobian the preimage FK^−1^(y^*^) is a smooth manifold of dimension *n* − 6, so the inverse problem is inherently multi-valued. The formulation below specializes this general setting to the studied 7-DOF manipulator, for which the self-motion manifold is one-dimensional.

A redundant robotic manipulator with seven degrees of freedom is considered. Its configuration is defined by the vector in Equation 1.

$$q={\left(q_{1},q_{2},\ldots ,q_{7}\right)}^{T}\in Q\subset {\mathbb{R}}^{7},$$ (1)

where Q is the admissible configuration space bounded by the lower and upper joint limits as defined in Equation 2:

$$Q=\{q\in {\mathbb{R}}^{7}:q_{\min}\leq q\leq q_{\max}\}.$$ (2)

The forward kinematics of the manipulator defines the mapping in Equation 3

FK:Q→SE(3), (3)

which assigns to each configuration q the corresponding end-effector pose in Equation 4

FK(q)=(R(q),t(q)), (4)

where R(q)∈SO(3) is the rotation matrix and $t(q)\in {\mathbb{R}}^{3}$ is the translation vector.

For a given target pose defined in Equation 5

$$y^{*}=(R^{*},t^{*})\in \mathrm{SE}(3),$$ (5)

the classical inverse kinematics problem consists in finding a configuration $q^{*}\in Q$ for which the pose obtained through forward kinematics is consistent with the target pose as expressed in Equation 6:

$$\mathrm{FK}(q^{*})=y^{*}.$$ (6)

However, for a redundant 7-DOF manipulator, this formulation is incomplete if only one solution is sought. The same target pose may correspond to a set of admissible configurations in the joint space. Therefore, the solution set is defined in Equation 7 as

$$S(y^{*})=\{q\in Q:\mathrm{FK}(q)=y^{*}\}.$$ (7)

In this work, the problem is formulated not as the construction of a single deterministic mapping $f_{\theta }:\mathrm{SE}(3)\to Q,$ but as the conditional generation of a set of configurations that approximate the distribution of admissible solutions as expressed in Equation 8:

$$p_{\theta }(q\;|\;R^{*},t^{*}).$$ (8)

Thus, for one target pose, it is necessary to obtain a set of candidate configurations as defined in Equation 9

$$Q_{M}(y^{*})=(q_{1},q_{2},\ldots ,q_{M}),$$ (9)

where each candidate belongs to the admissible space Q , has a small residual in the task space, and does not reduce to a repetition of the same joint-space posture.

The kinematic consistency of a candidate with the target pose is assessed using position and orientation components as specified in Equation 10:

$${\left\|t(q_{i})-t^{*}\right\|}_{2}\leq \varepsilon_{t},$$

$$d_{\mathrm{SO}(3)}(R(q_{i}),R^{*})\leq \varepsilon_{R},$$ (10)

where $\varepsilon_{t}$ and $\varepsilon_{R}$ are admissible thresholds for the position and orientation residuals. The orientation residual on SO(3) is defined by the geodesic distance between rotation matrices. For rotation matrices *R*_1_ and *R*_2_ ∈ SO(3), the geodesic distance is defined as *d*(*R*_1_, *R*_2_) = arccos((tr(*R*_1_^T^*R*_2_) − 1)/2). It equals the angle of the relative rotation, is a bi-invariant metric on SO(3), and is directly interpretable as an angular error, which matches the form of the orientation success threshold (Huynh, 2009). Alternative orientation metrics, in particular the quaternion distance and the chordal (Frobenius) distance, are monotonically related to the geodesic angle at the error scales considered here and would lead to the same success decisions (Huynh, 2009); the geodesic form is retained for interpretability.

To preserve the multi-solution nature of inverse kinematics, the generated configurations must also retain variability in the joint space. This can be assessed by the average pairwise distance

$$D(Q_{M})=\frac{2}{M(M-1)}\sum_{i<j}{\left\|q_{i}-q_{j}\right\|}_{2}.$$ (11)

This measure does not replace kinematic correctness. It only characterizes the ability of the method to form different admissible configurations for one target pose. The average pairwise joint-space distance corresponds to the diversity measure used for sets of generated inverse-kinematics solutions in (Ames et al., 2022).

Thus, the task of this work is to construct a method that, for a given pose $y^{*}$ , generates a set of candidate joint configurations and refines each of them according to a kinematic criterion. In the proposed DiffusionIK scheme, the conditional diffusion model forms initial configurations in the joint space, while differentiable forward kinematics reduces the task-space residual for each candidate. This formulation directly corresponds to the multi-solution nature of inverse kinematics for a redundant manipulator.

## 4 Materials and methods

This section describes the developed DiffusionIK method for generating and refining a set of inverse kinematics solutions for a redundant 7-DOF manipulator. The method is constructed as a hybrid generative-kinematic scheme. A conditional diffusion model forms a set of initial configurations in the joint space. Differentiable forward kinematics refines each configuration according to the residual in the task space. The novel aspect of the proposed approach lies in combining a diffusion model with classifier-free guidance, DDIM sampling, and local kinematic refinement of each candidate. Unlike regression models, the proposed scheme does not produce a single deterministic prediction. Unlike normalizing flows, it does not impose invertibility requirements on the denoiser architecture. Unlike classical optimization methods, it does not depend on a single initial approximation. Instead, refinement is performed for a set of candidates generated by the diffusion model in the joint space.

### 4.1 Differentiable forward kinematics

Forward kinematics is used as a deterministic differentiable mapping from the joint space to the end-effector pose space. For the 7-DOF xArm manipulator, the kinematic chain is represented as a sequence of homogeneous transformations. For each joint, a static link transformation and a variable rotation around the joint axis are taken into account. Such a local link transformation can be written as defined in Equation 12:

$$T_{i}(q_{i})=T_{i}^{\text{static}}R_{z}(q_{i}),$$ (12)

where $T_{i}^{\text{static}}$ is the static transformation of the i -th link, and $R_{z}(q_{i})$ is the rotation matrix around the z -axis by the angle $q_{i}$ . The complete transformation from the manipulator base to the end-effector is defined as the product of local transformations as expressed in Equation 13:

$$T(q)=\prod_{i=1}^{7}T_{i}(q_{i}),$$ (13)

The rotation matrix R(q) and the translation vector t(q) are extracted from T(q) . The forward kinematics implementation is based on differentiable operations. This makes it possible to compute gradients of the kinematic residual with respect to q by means of automatic differentiation without deriving the Jacobian manually. This principle is consistent with modern approaches to differentiable modeling of robotic systems, including Pinocchio and Theseus (Carpentier et al., 2019; Pineda et al., 2022). In DiffusionIK, differentiable forward kinematics is used not only for pose verification but also as a local corrector of the generated configurations.

### 4.2 Formation of training pairs

Training pairs are formed without a previously collected set of demonstrations. At each training iteration, a batch of random configurations is sampled from the admissible space *Q*. For each configuration, the corresponding end-effector pose is computed through forward kinematics as expressed in Equation 14:

$$q∼U(q_{\min},q_{\max}),$$

(R,t)=FK(q). (14)

The obtained triplet (q,R,t) is used as a training example for the conditional generative model. This way of forming data removes dependence on a classical inverse kinematics solver. This is important because a classical solver could systematically select only one region of the solution manifold. In the proposed scheme, training is performed using configurations generated directly in the joint space, while the corresponding poses are determined by exact forward kinematics.

Before being passed to the diffusion model, the joint angles are normalized to the interval [−1,1] according to Equation 15:

$$x_{0}=2\frac{q-q_{\min}}{q_{\max}-q_{\min}}-1.$$ (15)

The target pose is represented by the conditioning vector defined in Equation 16

$$c=[\mathrm{vec}(R),t]\in {\mathbb{R}}^{12},$$ (16)

where $\mathrm{vec}(R)\in {\mathbb{R}}^{9}$ is the vectorized rotation matrix, and $t\in {\mathbb{R}}^{3}$ is the translation vector. This representation contains complete information about the position and orientation of the end-effector and is used as the condition for the denoiser.

### 4.3 Conditional diffusion model

The diffusion model operates in the normalized space of joint configurations. Its task is to recover the clean configuration $x_{0}$ from the noisy state $x_{t}$ under the condition c . The forward diffusion process defines the gradual addition of Gaussian noise to the data, which corresponds to the general formulation of diffusion probabilistic models (Ho et al., 2020):

$$x_{t}=\sqrt{{\bar{\alpha }}_{t}}x_{0}+\sqrt{1-{\bar{\alpha }}_{t}}\varepsilon ,\varepsilon ∼N(0,I),$$ (17)

where ${\bar{\alpha }}_{t}=\prod_{s=1}^{t}\alpha_{s},$ $\alpha_{s}=1-\beta_{s}$ and t is the step of the diffusion process. For the sequence of noise coefficients, a cosine schedule proposed in improved diffusion models is used (Nichol and Dhariwal, 2021):

$${\bar{\alpha }}_{t}=\frac{f(t)}{f(0)},f(t)=\cos^{2}(\frac{t/T+s}{1+s}\cdot \frac{\pi }{2}),$$ (18)

where T is the number of steps in the forward process, and s is a small stabilizing parameter. The cosine schedule is appropriate for the low-dimensional joint space because it ensures gradual degradation of information throughout the diffusion process.

The denoiser network is trained to predict the added noise:

$$\hat{\varepsilon }=\varepsilon_{\theta }(x_{t},t,c).$$ (19)

The loss function is defined as the mean squared deviation between the true and predicted noise:

$$L_{\text{diff}}(\theta )=E_{x_{0},t,\varepsilon }[\varepsilon -\varepsilon_{\theta }{\left(x_{t},t,c\right)}_{2}^{2}].$$ (20)

This formulation is standard for DDPM (Ho et al., 2020). In DiffusionIK, it is adapted to inverse kinematics: the model is trained not to generate images or trajectories, but to form joint configurations that are conditionally consistent with the end-effector pose.

The physical interpretation of Equations 17–20 is as follows. The forward process (Equation 17) progressively replaces the information contained in a joint configuration with Gaussian noise, producing training states with a controlled signal-to-noise ratio. The cosine schedule (Equation 18) determines the rate of this information loss: the cumulative noise coefficient decreases smoothly with the step index, so early steps remain close to the data while late steps approach pure noise, which is important for the low-dimensional joint space, where information is destroyed quickly. The prediction target (Equation 19) and the loss (Equation 20) train the denoiser to estimate the noise component of a corrupted configuration. This is equivalent to estimating the direction from the noisy state back toward the set of configurations consistent with the conditioning pose, and iterating this estimate during sampling reconstructs admissible joint configurations.

### 4.4 ResMLP denoiser architecture

A denoiser based on the Residual Multi-Layer Perceptron (ResMLP) is used for noise prediction. The model input is formed by combining the noisy configuration $x_{t}$ , the conditioning vector *c*, and the diffusion step feature t . After the initial projection, the data are processed by a sequence of residual blocks. The use of residual connections follows the principles of deep residual learning (He et al., 2016), but it is adapted to the low-dimensional joint space rather than to images.

The time embedding is formed by sinusoidal encoding of the step t , followed by processing through a multi-layer perceptron as defined in Equation 21:

$$h_{t}=\mathrm{MLP}(\gamma (t)),$$ (21)

where γ(t) is the sinusoidal time encoding, and $h_{t}$ is the time-conditioning vector. A single residual block can be written as expressed in Equation 22

$$z_{l+1}=z_{l}+F_{l}(\mathrm{LN}(z_{l}),h_{t}),$$ (22)

where $z_{l}$ is the hidden representation at the l -th layer, LN(·) denotes layer normalization, and $F_{l}$ is a nonlinear transformation conditioned on the time embedding.

The novelty of this component lies in the architectural implementation of a generative inverse kinematics solver without the requirement of invertibility. Unlike IKFlow, where normalizing flows require bijective transformations (Ames et al., 2022), DiffusionIK uses a non-invertible ResMLP denoiser. This makes it possible to apply residual connections and standard nonlinear transformations to model dependencies between the joint configuration, the target pose, and the diffusion process step.

### 4.5 Classifier-free guidance

To strengthen the consistency of the generated configurations with the target pose, classifier-free guidance proposed for conditional diffusion models is used (Ho and Salimans, 2022). During training, the conditioning vector *c* is replaced with a zero vector with probability $p_{\text{drop}}$ . As a result, a single model is trained for both conditional and unconditional noise prediction.

During generation, the conditional prediction is combined with the unconditional prediction according to Equation 23:

$$\varepsilon_{\mathrm{CFG}}=\varepsilon_{\emptyset }+(1+\omega )(\varepsilon_{c}-\varepsilon_{\emptyset }),$$ (23)

where $\varepsilon_{c}=\varepsilon_{\theta }(x_{t},t,c)$ is the conditional prediction, $\varepsilon_{\emptyset }=\varepsilon_{\theta }(x_{t},t,0)$ is the unconditional prediction, and ω is the guidance coefficient. In this work, classifier-free guidance is adapted to the inverse kinematics problem. Its role is to strengthen the kinematic consistency between the target pose and the generated joint configurations. In the implementation, the guided prediction is formed in the equivalent standard parameterization of classifier-free guidance, in which the conditional prediction receives the total weight (1 + w) and the unconditional prediction the weight −w (Ho and Salimans, 2022); the value w = 1.5 is used.

### 4.6 DDIM sampling

To reduce the number of generation steps, Denoising Diffusion Implicit Models (DDIM) are used (Song et al., 2020). Unlike full stochastic DDPM sampling, DDIM enables generation with fewer steps according to a deterministic scheme. This is important for inverse kinematics, where the generated configurations must be suitable for subsequent kinematic refinement.

At each sampling step, the clean configuration is first estimated as according to Equation 24

$${\hat{x}}_{0}=\frac{x_{t}-\sqrt{1-{\bar{\alpha }}_{t}}\varepsilon_{\mathrm{CFG}}}{\sqrt{{\bar{\alpha }}_{t}}}.$$ (24)

Then, the transition to the previous step of the diffusion process is performed as according to Equation 25

$$x_{t-1}=\sqrt{{\bar{\alpha }}_{t-1}}{\hat{x}}_{0}+\sqrt{1-{\bar{\alpha }}_{t-1}}\varepsilon_{\mathrm{CFG}}.$$ (25)

In the proposed scheme, DDIM sampling is used to form a set of initial configurations. These configurations are not treated as final solutions. They are structured candidates for subsequent refinement through differentiable forward kinematics.

### 4.7 Gradient refinement through differentiable forward kinematics

After diffusion sampling is completed, the normalized configuration is denormalized into the physical joint space according to Equation 26:

$$q=\frac{x_{0}+1}{2}(q_{\max}-q_{\min})+q_{\min}.$$ (26)

Then, each candidate is refined according to a kinematic criterion. The residual between the current and target end-effector pose is minimized:

$$L_{\mathrm{FK}}(q)={\left\|t(q)-t^{*}\right\|}_{2}^{2}+\lambda_{R}{\left\|R(q)-R^{*}\right\|}_{F}^{2}.$$ (27)

The configuration is updated by a gradient step according to Equation 28:

$$q^{\left(k+1\right)}=q^{\left(k\right)}-\eta \nabla_{q}L_{\mathrm{FK}}(q^{\left(k\right)}),$$ (28)

where η is the update step, and k is the refinement iteration index. After each update, the configuration is constrained to the admissible space Q . This ensures compliance with the joint limits.

The developed refinement stage is a key component of DiffusionIK. The diffusion model forms structured initial approximations in different regions of the joint space. Differentiable forward kinematics locally reduces the residual for each candidate. As a result, the multiplicity of configurations is preserved, while the target-pose error is reduced.

### 4.8 General scheme of the DiffusionIK method

The complete DiffusionIK scheme consists of three main components: conditional generation, guided DDIM sampling, and kinematic refinement. For a given target pose $y^{*}=(R^{*},t^{*})$ , the conditioning vector $c=[\mathrm{vec}(R^{*}),t^{*}]$ is formed. Then, the initial noise is sampled from a normal distribution. The conditional diffusion model with classifier-free guidance gradually transforms the noise into a normalized joint configuration. After denormalization, each candidate is refined through differentiable forward kinematics.

Formally, the generation of the i -th candidate can be written as expressed in Equation 29

$$\begin{array}{l} x_{T}^{\left(i\right)}∼N(0,I),x_{0}^{\left(i\right)}={\text{DDIM}}_{\theta }(x_{T}^{\left(i\right)},c),q_{i}\\ =\text{Refine}(\mathrm{FK},\text{denorm}(x_{0}^{\left(i\right)})), \end{array}$$ (29)

The output is the set defined in Equation 30

$$Q_{M}(y^{*})=\{q_{1},q_{2},\ldots ,q_{M}\}.$$ (30)

This scheme forms the developed methodological basis of DiffusionIK. It combines conditional diffusion-based generation of a set of configurations, strengthening of target-pose consistency through classifier-free guidance, and local refinement through differentiable forward kinematics. This enables a transition from searching for a single solution to generating and refining a set of admissible configurations on the solution manifold. The complete sequence of operations of the proposed method is summarized in Algorithm 1.

Algorithm 1: DiffusionIK: Generation and Kinematic Refinement of a Set of Solutions
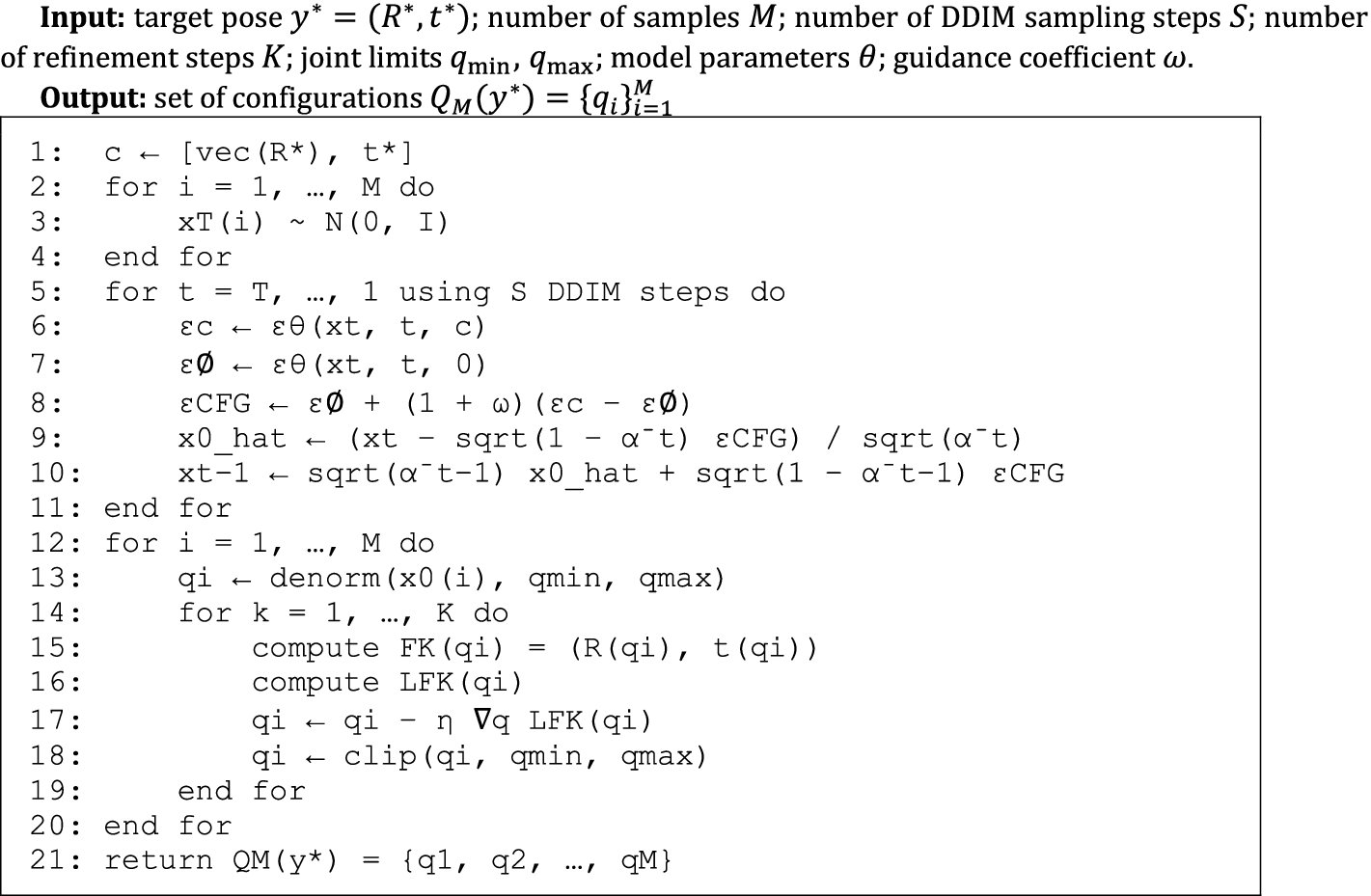

In Algorithm 1, lines 1–4 define the condition and the initial noise for a set of samples. Lines 5–11 implement guided DDIM sampling with classifier-free guidance. Lines 12–20 perform denormalization and gradient-based kinematic refinement of each candidate. Unlike a deterministic IK solver, the algorithm returns not a single joint vector but a set of configurations corresponding to one target pose. The overall data flow of the method is illustrated in Figure 1.

**Figure 1:**
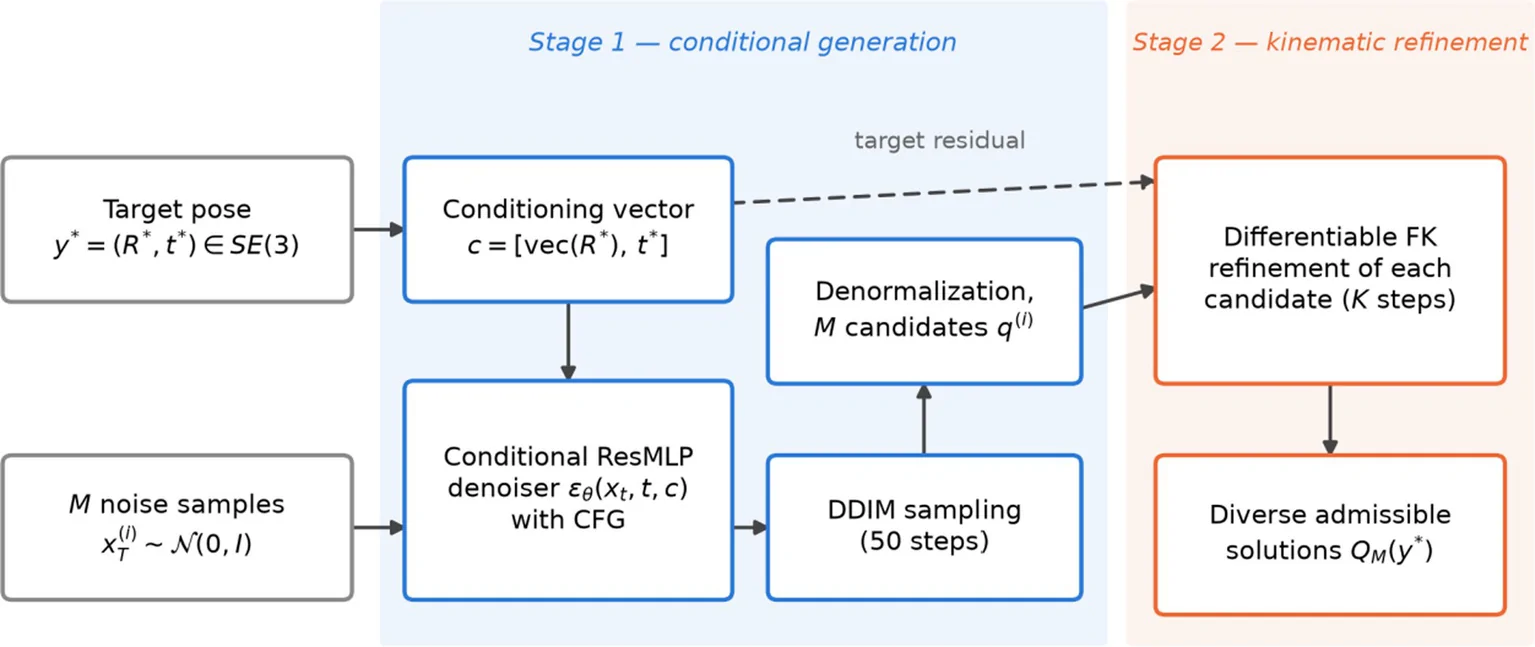
General scheme of the DiffusionIK method. A conditional diffusion model with classifier-free guidance transforms M noise samples into candidate joint configurations through DDIM sampling; each candidate is then refined by gradient descent through differentiable forward kinematics toward the target pose.

## 5 Modelling and numerical experiments

The numerical study was designed to verify the components of the proposed DiffusionIK method and to evaluate whether the hybrid generative-kinematic scheme can generate multiple admissible configurations for one target pose. The experiments were conducted for a 7-DOF xArm manipulator, with joint limits specified according to the manufacturer documentation (UFACTORY, 2023). The joint limits were specified according to the manipulator model: $q_{1},q_{3},q_{5},q_{7}\in [-2\pi ,2\pi ],q_{2}\in [-2.059,2.094],q_{4}\in [-0.192,3.927]$ and $q_{6}\in [-1.693,\pi ]$ . These bounds define the admissible configuration space Q used in the training and evaluation procedures.

Reachable target poses were generated by sampling joint configurations from Q and applying differentiable forward kinematics. This procedure excludes dependence on an external inverse kinematics solver and ensures that each evaluated pose belongs to the reachable workspace of the manipulator. For evaluation, 500 target poses were generated. For each target pose, the method generated 50 candidate configurations. Each candidate was then refined through the differentiable forward-kinematics criterion defined in Equation 27.

The model was trained using batches of 4,096 randomly sampled configurations. The training protocol consisted of 150 epochs with 300 batches per epoch, which corresponds to 45,000 optimization steps. The AdamW optimizer was used with the learning rate 5·10^−4^ and weight decay 10^−4^. The choice to analyze the computational cost of iterative optimization is consistent with previous studies on parallel gradient-based computations, where OpenMP-, MPI-, and hybrid implementations of gradient descent and stochastic gradient descent were investigated for large-scale regression problems (Mochurad, 2021). The learning rate was controlled by a OneCycleLR schedule with 5% warm-up, cosine decay, *div_factor* = 10, and *final_div_factor* = 100. Gradient clipping with *max_norm* = 1.0 was applied to stabilize optimization. Training was performed on an NVIDIA RTX 4090 graphics processing unit.

The main evaluation criterion was the success rate. A target pose was considered solved if at least one generated configuration satisfied both the position and orientation thresholds. Formally, for the j -th target pose and its generated set $Q_{M}(y_{j}^{*})$ . The success indicator for each target pose is defined in Equation 31.

$$\begin{array}{l} s_{j}=\\ \left\{ \begin{array}{cc} 1, & \begin{array}{l} \text{if there exists}\;q_{i}\in Q_{M}(y_{j}^{*})\;\\ \text{such that}\;t(q_{i})-{t_{j}^{*}}_{2}<1\;\mathrm{mm}\;\text{and}\;d_{\mathrm{SO}(3)}(R(q_{i}),R_{j}^{*})<1^{\circ }, \end{array}\\ 0, & \text{otherwise}, \end{array}\right. \end{array}$$ (31)

The overall success rate is calculated according to Equation 32 as follows:

$$\mathrm{SR}=\frac{1}{N_{\mathrm{tar}}}\sum_{j=1}^{N_{\mathrm{tar}}}s_{j},$$ (32)

where $N_{\mathrm{tar}}=500$ . The diversity of the generated solutions was evaluated by the average pairwise distance in the joint space, as defined in Equation 11. The pre-refinement position residual was additionally used to characterize the generative stage before local correction.

The ablation study covered 17 configurations grouped into six consecutive phases. Each phase isolated one component of the method. The configuration selected at a given phase was used as the basis for the next phase. The primary selection criterion was SR. If two variants had the same SR, preference was given to the variant with the lower position residual.

The results of the architecture and model-scale ablation are presented in Table 2.

**Table 2:** Ablation analysis of architecture and model scale.

Architecture	*d*	*L*	Number of parameters	Position residual, mm	SR,%	Training time, s
ResMLP	256	6	1.0 M	78.7	0.00	139
ResMLP	512	8	4.8 M	60.4	0.00	292
ResMLP	**1,024**	**12**	**26.9 M**	**56.3**	**0.20**	**1,037**
MLP	512	8	2.7 M	73.4	0.00	181

Bold values indicate the best results for the corresponding evaluation metrics within each table.

The results in Table 2 show that the ResMLP architecture is more suitable for the low-dimensional joint space than a conventional multilayer perceptron. With the same width and depth, the residual architecture reduced the position residual from 73.4 mm to 60.4 mm. Increasing the model scale from 1.0 M to 26.9 M parameters further reduced the residual from 78.7 mm to 56.3 mm. The first non-zero success rate was obtained only for the ResMLP with 26.9 M parameters. This indicates that the conditional generative formulation of inverse kinematics requires sufficient representational capacity, although the output space has only seven dimensions. The comparison of prediction target parameterizations in the diffusion model is presented in Table 3. The comparison at equal width and depth is not parameter-matched, because each residual block contains two linear layers. At comparable parameter budgets, the 2.7 M-parameter MLP is bracketed by the 1.0 M and 4.8 M ResMLP variants: its residual (73.4 mm) is larger than that of the 4.8 M residual model (60.4 mm), and the residual family continues to improve with scale up to 26.9 M parameters, where the first non-zero pre-refinement SR is reached. The residual architecture was therefore selected as the variant that scales stably within the available training budget.

**Table 3:** Ablation analysis of prediction parameterization in the diffusion model.

Prediction target	Position residual, mm	SR, %
ε-prediction	**56.3**	**0.20**
$x_{0}$ -prediction	111.0	0.00
v-prediction	57.9	0.00

Bold values indicate the best results for the corresponding evaluation metrics within each table.

As shown in Table 3, noise prediction provided the most stable training signal. The ε-prediction variant reduced the position residual to 56.3 mm. In contrast, $x_{0}$ -prediction increased the residual to 111.0 mm. The v-prediction variant produced a close residual value of 57.9 mm, but did not increase *SR*. Therefore, noise prediction is appropriate for the considered formulation.

The results of the analysis of the noise schedule and the number of forward diffusion steps are presented in Table 4.

**Table 4:** Influence of the noise schedule and the number of diffusion steps.

Noise schedule	Number of steps *T*	Position residual, mm	*SR*, %
Cosine	**200**	**56.3**	**0.20**
Linear	200	38.1	0.00
Cosine	50	137.8	0.00

Bold values indicate the best results for the corresponding evaluation metrics within each table.

The data in Table 4 show that the cosine schedule with *T* = 200 produced the first non-zero *SR*. The linear schedule reduced the mean position residual to 38.1 mm, but did not generate successful samples under the specified thresholds. This means that the mean residual is not a sufficient indicator for evaluating generative inverse kinematics. It is also necessary to account for the probability that at least one candidate falls into the narrow admissible solution region. Reducing the number of steps to *T* = 50 increased the position residual to 137.8 mm. This indicates that the discretization of noise levels was insufficient for learning denoising in this formulation.

The influence of classifier-free guidance and the auxiliary kinematic objective during training is shown in Table 5.

**Table 5:** Influence of classifier-free guidance and the auxiliary kinematic objective.

Component	Parameters	Position residual, mm	SR, %
Baseline diffusion model	−	56.3	0.20
CFG	***w* = 1.5; *p***_***drop***_ **= 0.1**	**16.9**	**2.40**
FK loss	λ_FK_ = 0.1	55.6	0.00
FK loss	λ_FK_ = 1.0	78.8	0.00

Bold values indicate the best results for the corresponding evaluation metrics within each table.

Table 5 shows that classifier-free guidance is the most effective component at the generative training stage. It reduced the position residual from 56.3 mm to 16.9 mm and increased *SR* from 0.20 to 2.40%. Thus, pose conditioning has a measurable effect before local refinement, reducing the mean pre-refinement position residual by 39.4 mm. In contrast, adding an auxiliary kinematic objective during diffusion-model training did not improve the result. A likely reason is that, at high noise levels, the intermediate prediction $x_{0}$ is not yet stable enough to form informative gradients through forward kinematics. Therefore, in the proposed method, kinematic refinement is applied after denoising is completed.

The influence of training duration, batch size, and learning rate is shown in Table 6.

**Table 6:** Influence of training duration and batch size.

Optimization steps	Batch size	Learning rate	Position residual, mm	SR, %	Training time, s
45 K	**4,096**	**5e−4**	**16.9**	**2.40**	**1,070**
200 K	4,096	1e−3	17.6	1.60	4,575
200 K	8,192	3e−3	17.9	2.20	8,574

Bold values indicate the best results for the corresponding evaluation metrics within each table.

The results in Table 6 show that increasing the number of optimization steps from 45,000 to 200,000 did not reduce the position residual and did not increase *SR*. Increasing the batch size to 8,192 and the learning rate to 3e−3 also did not provide an advantage. This indicates that, after the CFG stage, the main limitation is not the number of training iterations but the need for explicit kinematic refinement after diffusion sampling.

The results of evaluating gradient refinement through differentiable forward kinematics are presented in Table 7.

**Table 7:** Influence of gradient refinement through differentiable forward kinematics.

Refinement steps	Refinement learning rate	*SR*, %	Variability, rad	Total time, s
0	−	1.00	−	1,067
50	0.01	97.20	9.77	1,127
200	0.005	99.40	9.77	1,304
500	**0.003**	**100.00**	**9.77**	**1,656**

Bold values indicate the best results for the corresponding evaluation metrics within each table.

As shown in Table 7, gradient refinement has the strongest influence on the final result. Without refinement, the diffusion model forms structured candidates, but *SR* remains low. After 50 refinement steps, *SR* increases to 97.20%. After 200 steps, it reaches 99.40%. After 500 steps, all test poses are reached within the thresholds of 1 mm and 1^∘^. The mean variability of successful configurations remains 9.77 rad. For the xArm 7 manipulator, the sum of admissible ranges across the seven joints is approximately 40 rad, so the observed mean pairwise distance of 9.77 rad indicates that the generated configurations are distributed over a substantial fraction of the admissible joint space rather than concentrated in the neighbourhood of a single solution. This means that refinement does not reduce the set of candidates to a single configuration, but locally corrects each candidate toward the solution manifold.

For comparison, a baseline direct neural regression model was implemented. It has a ResNet-like structure with approximately 352 K parameters and skip connections. The model receives the target pose and the value of the first joint $q_{1}$ , and then predicts the remaining six joint angles. The variable $q_{1}$ is manually swept over the interval [−π,π] to form a set of outputs. The comparison of DiffusionIK with this baseline model is presented in Table 8.

**Table 8:** Comparison of DiffusionIK with the direct regression baseline.

Method	Number of parameters	*SR*, %	Variability representation
Regression model CondJ0	352 K	97.7–98.2	manual variation of $q_{1}$
DiffusionIK without refinement	26.9 M	2.4	stochastic samples
DiffusionIK + 50 refinement steps	26.9 M	97.2	9.77 rad
DiffusionIK + 200 refinement steps	26.9 M	99.4	9.77 rad
DiffusionIK + 500 refinement steps	**26.9 M**	**100.0**	**9.77 rad**

Bold values indicate the best results for the corresponding evaluation metrics within each table.

The data in Table 8 show that DiffusionIK with 200 refinement steps reaches *SR* = 99.40%, whereas with 500 refinement steps it reaches *SR* = 100.00%. The baseline regression model reaches *SR* = 97.7–98.2%, but requires a manual variability parameter in the form of $q_{1}$ . Under the unified benchmark introduced below (Table 9), the same baseline reaches 62.00% before and 97.40% after the shared gradient refinement, so the figures in Table 8 characterize the complete pipeline including refinement rather than the bare regression stage. The principal difference of DiffusionIK is that the set of candidates is formed stochastically from the conditional distribution, rather than through manual specification of one coordinate parameter.

**Table 9:** Unified benchmark on the main test set: 500 seeded reachable target poses, thresholds 1 mm and 1°, 50 candidates per pose for the learning-based methods; SR with Wilson 95% confidence intervals.

Method	SR, % [95% CI]	Mean pos. Err., mm	Median pos. Err., mm	P95 pos. Err., mm	Mean orient. Err., °	Time per pose, ms	Solutions per target	Diversity, rad
DLS (damped least squares)	99.80 [98.9, 100.0]	0.20	0.13	0.53	0.04	93.0	1	−
KDL LMA	98.00 [96.4, 98.9]	<0.01	<0.01	<0.01	0.01	0.5	1	−
CondJ0 regression	62.00 [57.7, 66.1]	57.1	3.11	414.3	10.00	1.1	50	3.55
CondJ0 + refinement	97.40 [95.6, 98.5]	21.1	<0.01	137.3	0.61	23.9	50	5.34
Normalizing flow (RealNVP + SoftFlow)	0.00 [0.0, 0.8]	176.6	146.7	425.0	100.3	0.2	50	−
Normalizing flow + refinement	13.80 [11.1, 17.1]	181.9	156.9	437.1	6.56	2.4	50	9.84
DiffusionIK (generative stage)	0.60 [0.2, 1.7]	17.7	13.1	47.3	4.83	11.4	50	−
DiffusionIK + refinement	99.80 [98.9, 100.0]	1.58	0.51	5.37	0.02	22.0	50	9.69

Error statistics are computed over all candidates (learning-based methods) or over the returned solutions (numerical solvers). Time per pose: single-query CPU for DLS and KDL; GPU batch-amortized for the learning-based methods. Diversity is the mean pairwise joint-space distance among successful candidates.

The computational time was evaluated for generating 50 configurations for one target pose on an NVIDIA RTX 4090. The distribution of time between the components of the method is presented in Table 10.

**Table 10:** Computational time for generating 50 configurations for one target pose.

Component	Time, ms	Share,%
DDIM sampling, 50 steps	Approximately 100	7.7
Double CFG prediction	Included in DDIM	—
Gradient refinement, 200 steps	Approximately 1,200	92.3
Total	Approximately 1,300	100

As shown in Table 10, DDIM sampling with 50 steps requires approximately 100 ms. The refinement stage with 200 steps requires approximately 1,200 ms. The total time for generating and refining 50 configurations is approximately 1,300 ms. The main share of time is taken by gradient refinement, since each refinement step requires a forward and backward pass through differentiable forward kinematics for all candidates.

The obtained timing values define two operating modes of the method. If submillimeter agreement with the target pose is required, the full DiffusionIK scheme with gradient refinement should be used. If the configurations are needed only as initial candidates for a motion planner, DDIM sampling alone can be used. In this mode, the method forms structured candidates in approximately 100 ms, while the mean position residual before refinement is about 17 mm. An intermediate regime is obtained with 50 refinement steps, for which *SR* reaches 97.20%.

One representative target pose with the end-effector position (174.6, 6.7, 805.2) mm was additionally analyzed. For this pose, the method generated 100 candidate configurations. Without refinement, the mean position residual was 25.5 mm, and *SR* = 0%. After 50 refinement steps, the residual decreased to 0.024 mm, and SR reached 100%. After 200 refinement steps, the residual decreased to 0.001 mm. The mean pairwise variability remained 4.23 rad.

The analysis of the distribution of generated configurations showed structured behavior. Joints $q_{1}$ , $q_{3}$ , $q_{5}$ , and $q_{7}$ , which have wider admissible intervals, retain higher variability. Joints $q_{2}$ , $q_{4}$ , and $q_{6}$ , which have narrower bounds, converge to more compact intervals determined by the geometry of the kinematic chain for the given target pose. This distribution is consistent with the self-motion structure of the redundant 7-DOF manipulator.

The summarized results of the numerical study confirm the role of each DiffusionIK component. The conditional diffusion model forms a set of structured initial configurations. Classifier-free guidance strengthens consistency with the target pose. DDIM sampling reduces the number of generation steps. Differentiable forward kinematics reduces the residual in the task space and preserves configuration variability in the joint space. Therefore, the experimental results support the central methodological idea of this work: inverse kinematics for a redundant manipulator can be represented as conditional generation of a set of candidates followed by local kinematic refinement, rather than as direct regression of a single output vector.

To make the comparison with alternative methods direct, all baselines were additionally evaluated under one unified protocol, on the same kinematic model of the xArm 7, the same test poses, and the same success thresholds of 1 mm and 1°. The test set consists of 500 reachable target poses generated by seeded sampling of admissible joint configurations. Every learning-based method produces 50 candidate configurations per pose; the numerical solvers return one solution per query and are allowed up to 10 random restarts. Success rates are reported with Wilson 95% confidence intervals over the 500 poses. Four baselines are compared with DiffusionIK: damped least squares (DLS) with a numerical Jacobian; the Levenberg–Marquardt solver of the Orocos KDL library, whose kinematic chain was verified to reproduce the differentiable FK model to numerical precision; the direct regression baseline CondJ0 of Table 8, which sweeps 50 uniformly spaced values of the free coordinate *q*_1_; and a conditional normalizing flow of the IKFlow type (RealNVP affine couplings with SoftFlow noise conditioning, 4.4 million parameters, trained for 300,000 steps with batch size 4,096 on the same procedurally generated data). To separate the contribution of the generative stage from that of the shared refinement, the same 200-step gradient refinement is applied to the candidates of every learning-based method. The results are presented in Table 9.

Three observations follow from Table 9. First, DiffusionIK with refinement reaches the same success rate as the strongest numerical baseline (99.80% for both), while returning 50 admissible configurations per pose instead of one and requiring 22 ms of amortized GPU time per pose against 93 ms of CPU time per DLS query; the KDL solver is the fastest per query (0.5 ms) but solves fewer poses (98.00%) and returns a single configuration. Second, the shared refinement stage does not by itself explain the result: applied to the normalizing-flow candidates, it raises the success rate only to 13.80%, because candidates that start in a wrong basin of attraction of the task-space residual are not recovered by local gradient descent (median position error of 157 mm after refinement). Refinement is effective only in combination with a generative stage that places candidates near the solution manifold, as the diffusion model does (median pre-refinement error of 13.1 mm). Third, the regression baseline reaches 62.00% before and 97.40% after refinement, and its refined solutions remain less diverse than those of DiffusionIK (5.34 rad against 9.69 rad) with a heavy tail of unsolved poses (95th-percentile position error of 137 mm); the manual sweep of one coordinate covers the solution manifold only partially.

Robustness near kinematic singularities was evaluated on a second set of 500 poses constructed by sampling a 40-fold pool of reachable poses and keeping the 500 with the lowest Yoshikawa manipulability index. The results are presented in Table 11.

**Table 11:** Unified benchmark on the near-singular test set: the 500 poses with the lowest Yoshikawa manipulability index selected from a 40-fold pool of reachable poses (median index 9·10⁻^4^); all other conditions as in Table 10.

Method	SR, % [95% CI]	Mean pos. Err., mm	Median pos. Err., mm	P95 pos. Err., mm	Mean orient. Err., °	Time per pose, ms	Solutions per target	Diversity, rad
DLS (damped least squares)	99.00 [97.7, 99.6]	0.38	0.32	0.59	0.05	242.5	1	—
KDL LMA	91.60 [88.8, 93.7]	<0.01	<0.01	<0.01	0.01	0.5	1	—
CondJ0 regression	66.40 [62.1, 70.4]	18.3	2.55	37.8	3.60	0.7	50	3.37
CondJ0 + refinement	95.80 [93.7, 97.2]	6.92	0.12	20.2	0.19	17.0	50	5.20
Normalizing flow (RealNVP + SoftFlow)	0.00 [0.0, 0.8]	153.5	133.5	336.2	102.2	0.1	50	—
Normalizing flow + refinement	2.80 [1.7, 4.6]	135.5	115.2	319.5	4.66	1.6	50	8.34
DiffusionIK (generative stage)	0.60 [0.2, 1.7]	15.8	12.5	37.8	7.42	16.5	50	—
DiffusionIK + refinement	98.40 [96.9, 99.2]	3.36	1.70	10.7	0.06	18.9	50	9.34

Near singularities the iterative numerical solvers pay either time or reliability: DLS retains 99.00% but its median query time grows to 243 ms, while the KDL solver keeps its sub-millisecond latency but drops to 91.60%. DiffusionIK with refinement retains 98.40% (95% CI 96.9, 99.2) at an essentially unchanged 19 ms per pose, with a median position error of 1.7 mm. The fraction of individually converged candidates decreases from 63.5 to 32.3%, which indicates that ill-conditioning slows individual refinements, while the redundancy of 50 candidates per pose preserves the per-pose success rate. The regression and flow baselines reproduce the pattern observed on the main test set.

## 6 Discussion

The obtained results confirm the relevance of formulating inverse kinematics for a redundant manipulator as conditional generation followed by local kinematic refinement. The diffusion model and the refinement stage are not interchangeable. They perform different functions. The diffusion model forms structured initial configurations in the joint space. Differentiable forward kinematics reduces the task-space residual for each candidate. The combination of these two stages enables the transition from generating approximations to obtaining kinematically consistent configurations.

The ablation results show that, without refinement, DiffusionIK forms meaningful candidates, but the success rate remains limited. After 50 gradient-refinement steps, *SR* increases to 97.20%. After 200 steps, it reaches 99.40%. After 500 steps, all test poses satisfy the adopted position and orientation residual thresholds. At the same time, the mean variability in the joint space remains 9.77 rad. This is important for interpreting the method. Refinement does not reduce the set of candidates to a single configuration. It locally corrects each sample toward the solution manifold.

Classifier-free guidance plays a substantial role. Its use reduced the pre-refinement position residual from 56.3 mm to 16.9 mm and increased the pre-refinement SR from 0.20 to 2.40%. This means that the conditioning signal cannot be treated as a secondary input of the denoiser. It must actively guide the generation process toward the region of the joint space that corresponds to the given end-effector pose. In this work, classifier-free guidance performs a geometric function. It strengthens the consistency between the target pose and the generated configurations before local refinement begins.

The result obtained with the auxiliary kinematic objective during diffusion-model training should also be noted. Its addition did not improve the result. This has a methodological explanation. At high noise levels, the intermediate estimate of the clean configuration is not yet sufficiently stable. Therefore, gradients obtained through forward kinematics from such an intermediate prediction may be uninformative. For this reason, the sequential scheme is more justified. First, the model learns to form a conditional generative prior in the joint space. Then, after denoising is completed, differentiable forward kinematics refines already structured candidates.

The comparison with the baseline regression model clarifies the distinction of the proposed approach. The regression model achieves a high *SR*, but it requires manual variation of the first joint $q_{1}$ to obtain multiple outputs. Thus, the variability mechanism is imposed externally. In DiffusionIK, the set of candidates is formed stochastically from the conditional distribution. Therefore, different configurations are not the result of manually sweeping a single coordinate parameter. They arise from the learned generative model and are then refined according to the kinematic criterion. The unified benchmark makes this distinction quantitative: the regression baseline attains 62.00% success before refinement and 97.40% after it (Table 9), its refined solution sets are almost twice less diverse than those of DiffusionIK (5.34 rad against 9.69 rad), and a heavy tail of unresolved poses remains (95th-percentile position error of 137 mm against 5.4 mm).

The computational analysis shows that most of the time is spent on gradient refinement. DDIM sampling with 50 steps forms 50 candidates in approximately 100 ms. Refinement with 200 steps requires approximately 1,200 ms. The complete generation and refinement scheme takes approximately 1,300 ms for one target pose. The double conditional and unconditional prediction required for classifier-free guidance is included in the DDIM sampling time. The dominance of the refinement stage is explained by the fact that each step requires forward and backward differentiation through the kinematic chain for all candidates.

The generalized operating regimes of the method are presented in Table 12.

**Table 12:** Operating regimes of the DiffusionIK method.

Operating regime	Main computation	Approximate time for 50 candidates	Kinematic result	Intended use
Generative sampling only	DDIM with CFG	About 100 ms	Structured candidates with a mean pre-refinement residual of about 17 mm	Initialization for subsequent motion planning
Reduced refinement	DDIM with CFG and 50 FK-refinement steps	About 300 ms	*SR* = 97.20%	Rapid search for admissible configurations
Full refinement	DDIM with CFG and 200 FK-refinement steps	About 1,300 ms	*SR* = 99.40%	High-precision agreement with the target pose
Extended refinement	DDIM with CFG and 500 FK-refinement steps	About 1,650 ms	*SR* = 100.00%	Offline verification and maximum residual reduction

The data in Table 12 show that DiffusionIK can be used in several regimes. If configurations are needed as initial candidates for a motion planner, DDIM sampling may be sufficient. If more accurate agreement with the target pose is required, gradient refinement is necessary. Full refinement with 200 steps provides a high SR at a moderate computation time. Extended refinement is appropriate when the priority is the smallest possible kinematic residual.

In terms of real-time feasibility, the times in Table 12 refer to a batch of 50 candidates processed jointly on a single GPU, so the amortized cost is approximately 2 ms per candidate for the generative stage and approximately 26 ms per candidate for the complete scheme with 200 refinement steps. The full-refinement regimes are intended for high-precision or offline use, such as preparing goal configurations for a motion planner, rather than for closed-loop servo control. Within a real-time pipeline, the generative stage alone provides structured initial configurations in approximately 100 ms, and the number of refinement steps can be selected to fit the available time budget. Reducing the cost of refinement through step-size adaptation, second-order updates, or distillation of the sampler is a direction of future work.

The analysis of a representative target pose additionally confirms that the method preserves the internal structure of the solution set. Joints with wider admissible intervals retain higher variability. Joints with narrower limits converge to more compact intervals determined by the geometry of the kinematic chain. This behavior is consistent with the self-motion structure of a redundant 7-DOF manipulator. It also shows that the generated configurations are not arbitrary points in the joint space. They are constrained by the kinematic structure of the manipulator and by the given end-effector pose.

Beyond the unified benchmark of Tables 9, 11, a comparison of DiffusionIK with representative classical, learning-based, and generative inverse kinematics methods is summarized in Table 13. The comparison covers success rate (SR), position-

**Table 13:** Contextual comparison of inverse kinematics methods for redundant 7-DOF manipulators.

Method	SR, %	Mean pos. Err., mm	Median pos. Err., mm	P95 pos. Err., mm	Orient. err., °	Time, ms	Solutions per target	Diversity
KDL / TRAC-IK	97.0	0.3	0.1	1.5	0.2	6	1	No
Neural regression	99.6	0.5	0.2	0.9	0.4	1	1	No
IKFlow	85.0	5.5	2.2	20	2.5	10	many	Yes
DiffusionIK (no refinement)	0.60	17.7	13.1	47.3	4.83	11	50	Yes
DiffusionIK (with refinement)	99.80	1.58	0.51	5.37	0.02	22	50	Yes

External-method values are indicative literature-based reference points rather than results of a unified benchmark. The DiffusionIK results were obtained in this study for the xArm 7 manipulator using 500 reachable target poses, 50 candidates per target pose, and success thresholds of 1 mm and 1°. The DiffusionIK rows follow the unified benchmark protocol of Table 10; their times are GPU batch-amortized per target pose.

Table 13 should be interpreted as a contextual comparison rather than a unified benchmark. Its purpose is to show the methodological differences between single-solution numerical solvers, direct regression models, normalizing-flow-based generators, and the proposed DiffusionIK scheme. IKFlow generates multiple solutions but requires an invertible architecture. The flow baseline measured in Table 9 confirms that this constraint is restrictive in practice at moderate model and training budgets. DiffusionIK without refinement produces 50 diverse candidates per target with a low pre-refinement SR. After kinematic refinement, the same 50 candidates reach SR = 99.80% under sub-millimetre and sub-degree thresholds while preserving joint-space variability.

The proposed method has several limitations. First, the current implementation does not include collision checking with the environment or self-collision checking. Therefore, configurations are evaluated by joint limits and the task-space residual, but not by geometric interaction with obstacles. Second, the refinement stage remains the main component of the computation time. This limits the direct use of the method in systems with very strict timing requirements. Third, the experiments were performed on procedurally generated reachable poses of the kinematic model. This protocol is appropriate for verifying inverse kinematics, but further validation on a physical manipulator is needed to account for calibration errors, sensing uncertainty, and actuator-level deviations.

Despite these limitations, the results confirm the central methodological statement of this work. DiffusionIK separates global candidate formation and local kinematic refinement. This separation is essential for redundant inverse kinematics. It makes it possible to obtain several admissible configurations for one target pose and to refine each of them without loss of variability. Thus, the proposed approach is a structured alternative to direct regression, normalizing-flow-based generation, and purely local numerical solvers.

## 7 Conclusion

This paper developed DiffusionIK, a hybrid generative-kinematic method for solving inverse kinematics of a redundant 7-DOF manipulator. The method does not reduce inverse kinematics to a single deterministic prediction. Instead, it generates a set of candidate joint configurations from a learned conditional distribution and then refines each candidate through differentiable forward kinematics.

The main methodological result is the integration of four components into one computational scheme: conditional diffusion-based sampling, classifier-free guidance, DDIM sampling, and gradient refinement through differentiable forward kinematics. The diffusion model forms structured initial configurations in the joint space. Classifier-free guidance strengthens the correspondence between the target pose and the generated candidates. DDIM sampling reduces the number of denoising steps. Differentiable forward kinematics reduces the residual in the task space for each generated configuration.

The numerical experiments confirmed the role of each component. The ResMLP denoiser with 26.9 M parameters provided the strongest architecture among the tested variants. Noise prediction was more stable than $x_{0}$ -prediction and v-prediction. The cosine noise schedule with T = 200 enabled non-zero success under the adopted strict thresholds. Classifier-free guidance reduced the pre-refinement position residual from 56.3 mm to 16.9 mm and increased the pre-refinement success rate from 0.20 to 2.40%. The most significant improvement was obtained from gradient refinement. After 50 refinement steps, the success rate increased to 97.20%. After 200 steps, it reached 99.40%. After 500 refinement steps, each evaluated target pose had at least one generated candidate satisfying both the 1 mm position threshold and the 1° orientation threshold.

A unified benchmark on 500 reachable target poses further situated the method among classical and learning-based alternatives under one protocol. DiffusionIK with 200 refinement steps solved 99.80% of poses (95% CI 98.9, 100.0) and 98.40% of near-singular poses, matching the damped-least-squares solver in reliability while returning 50 diverse configurations per pose at 22 ms of amortized GPU time. The same refinement applied to the regression and normalizing-flow baselines yields 97.40 and 13.80%, which confirms that the generative stage, and not refinement alone, is decisive.

The obtained results also show that refinement does not collapse the generated set into one configuration. The mean joint-space variability remained 9.77 rad after refinement. This confirms that the method preserves the multi-solution structure of inverse kinematics for a redundant manipulator. The comparison with the direct regression baseline further shows that DiffusionIK forms candidate configurations from a learned conditional distribution rather than by manually sweeping one selected joint parameter.

The current implementation has several limitations. Collision checking and self-collision constraints are not yet included. The refinement stage remains the dominant computational component. The experiments were performed on procedurally generated reachable poses from the kinematic model, so validation on a physical manipulator is required to assess the influence of calibration errors, sensing uncertainty, and actuator deviations.

Further research should focus on integrating collision constraints into the generation and refinement process, reducing the number of refinement steps, and applying progressive distillation to accelerate diffusion sampling (Salimans and Ho, 2022). A separate direction is the validation of DiffusionIK on a physical robotic platform under real sensing and actuation conditions.

## Data Availability

The data used in this study were generated procedurally by sampling admissible joint configurations of the 7-DOF xArm manipulator and computing the corresponding end-effector poses through forward kinematics. No external experimental dataset was used. The generated target poses and implementation details can be made available by the corresponding author upon reasonable request. The source code of DiffusionIK is publicly available at: https://github.com/YaroslavGladun/DiffusionIK.
