# Supplementary material for: Conditional diffusion-based inverse kinematics for redundant 7-DOF manipulators with differentiable refinement
Source: Front Artif Intell. 2026 Oct 16;9:1884749. doi: 10.3389/frai.2026.1884749 (PMC13719613; doi:10.3389/frai.2026.1884749)
Supplement: Supplementary file 1 [file Supplementary_file_1.docx]

# Appendix A. Architecture, Training, and Normalization Details

This appendix specifies the ResMLP denoiser, the training hyperparameters, and the input normalization used in DiffusionIK to make the reported results reproducible.

## A.1. ResMLP denoiser

The denoiser follows the layer specification in Table A1. The conditioning vector is injected once, by concatenation with the noisy normalized configuration at the input projection. The time embedding is injected additively inside every residual block through a block-specific linear projection applied after the first nonlinearity. No attention layers are used.

**Table A1.** Layer specification of the ResMLP denoiser (26,887,431 trainable parameters).

| **Component** | **Operation** | **Output size** |
| --- | --- | --- |
| Input assembly | concatenation of the noisy normalized configuration (7) and the conditioning vector (12) | 19 |
| Input projection | Linear 19 → 1024 | 1024 |
| Time embedding | sinusoidal encoding of the step t (128) → Linear 128 → 128 → SiLU → Linear 128 → 128 | 128 |
| Residual block, repeated 12 times | h = SiLU(Linear(LayerNorm(x))) + Linear_t(time embedding); x = x + Linear(LayerNorm(h)); all Linear layers 1024 → 1024, Linear_t: 128 → 1024 | 1024 |
| Output head | LayerNorm → Linear 1024 → 7 | 7 |

## A.2. Training hyperparameters

All hyperparameters of the training and sampling procedures are listed in Table A2. The guided prediction is computed in the standard classifier-free-guidance parameterization: with guidance weight w, the conditional prediction receives the total weight (1 + w) and the unconditional prediction the weight −w (Ho and Salimans 2022).

**Table A2.** Training, sampling, and refinement hyperparameters of DiffusionIK.

| **Parameter** | **Value** |
| --- | --- |
| Diffusion steps T; schedule | 200; cosine (s = 0.008) |
| Prediction target | noise (ε-prediction) |
| DDIM sampling steps | 50 (deterministic) |
| Classifier-free guidance | w = 1.5; condition dropout probability 0.1 (condition replaced by a zero vector) |
| Optimizer | AdamW, learning rate 5·10⁻⁴, weight decay 10⁻⁴ |
| Learning-rate schedule | OneCycleLR: 5% warm-up, cosine annealing, div_factor = 10, final_div_factor = 100 |
| Batch size and duration | 4096; 150 epochs × 300 batches = 45,000 optimization steps |
| Gradient clipping | global norm 1.0 |
| Training data | generated procedurally on the fly: joint configurations sampled uniformly within the joint limits, poses computed by forward kinematics; fresh samples in every batch |
| Refinement | Adam over joint variables; objective MSE(t) + MSE(R); (steps, learning rate) = (50, 0.01), (200, 0.005), (500, 0.003); joint limits enforced on the final configurations |
| Evaluation protocol | 500 reachable target poses × 50 candidates; success thresholds 1 mm and 1° |
| Hardware and framework | NVIDIA RTX 4090; PyTorch |

## A.3. Input normalization

Joint angles are normalized componentwise to [−1, 1] by the linear map x = 2(*q* − *q*_min_)/(*q*_max_ − *q*_min_) − 1, and generated samples are mapped back by the inverse transform with clipping to the joint limits. The conditioning vector c = [vec(R*), t*] is used without additional scaling: the nine elements of the rotation matrix are bounded in [−1, 1] by construction, and the translation components, expressed in meters, are bounded in magnitude by the manipulator reach (below 1.2 m for the xArm 7), which keeps all conditioning components on a comparable scale.
